# Highlights of Miami winter symposium 2015: into the era of immunotherapy

**DOI:** 10.7497/j.issn.2095-3941.2015.0008

**Published:** 2015-03

**Authors:** Wen Zhou

**Affiliations:** Department of Biological Sciences, Columbia University, New York, NY 10027, USA

**Keywords:** Personalized cancer medicine, immune therapy, immune checkpoint pathway, CTLA-4, PD-1, PD-L1

## Abstract

Personalized cancer medicine has seen significant improvements over the past decade. Recent Elsevier conference: Miami winter symposium 2015 (MWS2015) “Towards Personalized Cancer Medicine” meeting was dedicated to this exciting field, and focused on new progress in personalized drug development and antibody drug against checkpoint pathway. This meeting report summarizes the key developments presented and discussed at the meeting, with a focus on immunotherapy, especially on the CTLA-4 and PD-1/PD-L1 pathways. The monoclonal antibody drugs intervening these checkpoint pathways have the potential to play a larger role in personalize medicine within the near future. Here we intended to provide a comprehensive summary about ongoing trends and future perspectives on personalized medicine in cancer therapy.

The idea of personalized cancer medicine has stayed at the concept stage until recently. With the exciting progress on deep sequencing technology, personalized medicine receives more and more attention. To embrace this new exciting trend, the Elsevier conference: 2015 Miami winter symposium, entitled “Towards the Personalized Cancer Medicine” convened from January 18^th^ through January 21^st^, 2015 at Miami, FL.

The four-day scientific content covered basic cancer research and a variety of novel or emerging therapeutic approaches. The meeting consisted of six scientific sessions that included plenary talks given by twenty-five top-level leaders as well as three young investigators from both academia and industry. The subject material was diverse and included cancer heterogeneity, genetics, epigenetics, molecular mechanisms, targeted therapies, immune therapies and translational/clinical research. Exciting progress was reported on designing next-generation targeted therapy drugs, including inhibitors for BRD4, proteasome, deubiquitylase, and histone deacetylase. Perhaps the best feature of the meeting was the young investigator talks on the third day, in which trainees gave compelling fifteen-minute talks. Dr. Xiaoxia Zhu from the University of Miami presented a computational biochemical approach to investigate novel inhibitors of notch signaling pathway. Drs. Azzam and Bhang talked about drug screening in ovarian cancer and lentivirus-mediated cell barcoding technique to study cancer cell clonal dynamics, respectively.

Finally, in addition to the continuous success in chemical drugs, designing antibody drugs to inhibit tumor growth and to restore immunity was another hot topic in the meeting. Albeit tumor escaping immune surveillance by inhibiting immune checkpoint pathway has been known for years, and targeting this pathway to treat cancer has stagnated in clinic. One checkpoint protein is PD-1, which recruits a phosphatase and may interfere with T cell antigen receptor mediated signaling. Dendritic cells express PD-1 ligases, PD-L1 and PD-L2. Many tumor cells also express PD-L1, and its subsequent interaction with PD-1 on T cells is demonstrated to be a major mechanism of losing T cell immunity. Many pharmaceutics have developed PD-1 and PD-L1 antibodies, such as pembrolizumab (Merck), MPDL3280A (Genentech/Roche), nivolumab (Bristol-Myers Squibb) and MEDI4736 (Medimmune). These antibody drugs have shown responses against cancers of the lung, kidney, skin, bladder, head and necks. Some lymphomas showed response rates of 25% to 30% in clinical trials. Notably, FDA approved recently pembrolizumab for treatment in refractory melanoma in 2014.

CTLA-4 is another well-characterized checkpoint protein. CTLA-4 inhibited T cell activation by interfering T cell protein CD28 binding to its cognate ligands B7-1 and B7-2 on antigen presenting cells. FDA approved CTLA-4 antibody ipilimumab to treat metastatic melanoma in 2011. Studies since then have proven CTLA-4 antibodies’ efficacy against a broad range of cancer types, yet revealed its severe adverse effects. An encouraging progress was a recent phase II trial of the combination of anti-CTLA-4 and anti-PD-1 in melanoma showed a response rate of 50% where otherwise very limited treatment choice is available. Synergistically using two antibody drugs achieved a better response rate while reduced dose-related side effects.

These findings presented at the meeting validated the importance of personalized cancer therapy, especially on drug discovery and immune therapy. The overall success of personalized cancer therapy will heavily rely on getting the better of the expertise from all related fields, as well as fostering younger generation of scientists.

